# The Effect of H_2_O and CO_2_ on the Adsorption Behavior of H_2_ and CO on Hematite

**DOI:** 10.3390/ma18174175

**Published:** 2025-09-05

**Authors:** Xudong Mao, Baoqing Zhou, Hui Deng, Qiong Zeng, Jingbo Li, Jie Chen, Yiyu Xiao, Kuochih Chou

**Affiliations:** 1 Jiangxi General Institute of Testing and Certification, Nanchang 330052, China; 2 School of Resources & Environment, Nanchang University, Nanchang 330031, China; 3 State Key Laboratory of Advanced Metallurgy, University of Science and Technology Beijing, Beijing 100083, China

**Keywords:** density functional theory, adsorption behavior, carbon monoxide, hydrogen

## Abstract

The adsorption of gas reactant molecules (H_2_, CO, etc.) to the surface of hematite is the premise of chemical reaction. In order to further promote the basic research on the reaction mechanism of hematite reduction by a H_2_-CO gas mixture, the adsorption behavior of H_2_ (or CO) under the conditions of pre-adsorbed H_2_O (or CO_2_) was systematically studied by the density functional theory (DFT) combined with reduction experiments. The results indicate that the gas molecules (H_2_, CO, H_2_O and CO_2_) adsorbed on the Fe atom of the Fe_2_O_3_ (001) surface rather than the O atom, and the adsorption energy of the Fe_2_O_3_-CO adsorption system was relatively minimum (−1.317 eV), indicating that the Fe_2_O_3_-CO adsorption system was more stable. In addition, the adsorption energy of the H_2_ molecule adsorbed to the Fe_2_O_3_-H_2_O adsorption system was −0.132 eV, which was smaller than that of the H_2_ molecule directly adsorbed to Fe_2_O_3_ (−0.013 eV), indicating that the H_2_O molecule pre-adsorption was beneficial to the H_2_ molecule adsorption. Compared with the H_2_O molecule, the CO_2_ molecule had relatively less influence on the adsorption and subsequent behavior of CO with Fe_2_O_3_. From the experiment analysis results, on the whole, CO_2_ had a greater impact on the gas diffusion, while H_2_O had a greater impact on the interfacial chemical reaction (gas adsorption), which was consistent with the DFT calculation results.

## 1. Introduction

The iron industry is a globally important and fundamental industry, and the energy utilization structure of its production process poses a serious challenge to the environment [1]. The energy resource of the mainstream blast furnace–basic oxygen furnace (BF-BOF) route for ironmaking relies mostly on fossil fuel such as furnace coke and pulverized coal (mainly carbonaceous energy) [2,3]. The combustion of fossil fuel provides high-temperature heat and reducing agents, which is inevitably accompanied with huge CO_2_ (carbon dioxide) emissions [4,5,6]. Excessive emissions of the greenhouse gas CO_2_ have exacerbated global climate change, ocean acidification and other systemic environmental crises. As a result, the promotion of carbon-reducing transformation of the iron industry has become an urgent imperative [7]. Currently, it is difficult to completely replace blast furnaces for ironmaking. Therefore, the use of a H_2_-CO (hydrogen–carbon monoxide) gas mixture as a reducing agent to replace part of furnace coke for ironmaking is regarded as an important technology due to its significant reduction of direct CO_2_ emissions [8,9].

Understanding the reaction behavior and mechanism of the iron ore reduction by a H_2_-CO gas mixture is crucial for establishing a fundamental scientific basis for a green and efficient ironmaking industry [10,11]. Turkdogan et al. [12] observed that when reducing low-porosity hematite samples including synthetic oxides with a H_2_-CO gas mixture, there was no significant difference in the reduction process of different samples, and relevant studies [13,14,15] emphasized that partial alkaline oxides can enhance the reduction efficiency of iron oxides by increasing pellet porosity and changing the reaction path at a high temperature. Lyu et al. [16], using a thermo-gravimetric analyzer (800–1100 °C, 30%vol H_2_, 20 sccm), identified temperature as critical for improving the final reduction degree of the solid sample, and Zakeri et al. [17] confirmed that increasing H_2_ content in a H_2_-CO gas mixture would rapidly accelerate the reduction rate. In terms of the reduction mechanism, Jozwiak et al. [18,19] established that the reduction of hematite to iron was not a one-step reduction process. Hammam et al. [20] investigated the H_2_-based reduction process of compacts prepared from iron oxide using a thermo-gravimetric analyzer combined with a fitting model at 700–1100 °C, in which the results showed that the initial stage of the reduction process was governed by the chemical reaction kinetics, transitioning to gaseous diffusion control in the latter stage. Spreiter et al. [21] also concluded that the rate-controlling step in the iron ore reduction process would change with the change in experimental conditions and processes. In addition, Guo et al. [15,22] deduced from the reduction of iron oxide by H_2_ that the reduction process possessed the dissociation and adsorption of H_2_ on vacant sites on the iron oxide surface accompanied by the formation of hydroxide ions due to the release of oxygen from the lattice and eventually generated water molecules, and found that the FeO-Fe step was the rate-controlling step in the reduction reaction. Current research achievements primarily focus on the macroscopic scale of iron oxide reduction, while relatively few studies have been conducted at the microscopic scale (molecular) [23,24], indicating that the basic research on the molecular scale needs to be further strengthened.

At the molecular level, the chemical reaction between a H_2_-CO gas mixture and hematite can be roughly divided into three stages: gas adsorption, interface reaction and gas desorption. Crucially, the adsorption of gas reactant molecules (H_2_, CO, etc.) to the surface of hematite is the premise of the chemical reaction. Because of the advantage of density functional theory (DFT) in analyzing the adsorption behavior of a gas molecule on a surface [25,26], DFT calculations have been used to explore the adsorption behavior of a single gas molecule on the surface of iron oxide [27,28]. However, when H_2_ (or CO) reacts with hematite, H_2_O (or CO_2_) will be generated, and the generated H_2_O (or CO_2_) may continue to adsorb to the reaction active sites on the hematite surface to affect the reaction [23,29,30,31]. The effect of the reaction product re-adsorption situation has been relatively less studied. Therefore, in this paper, the adsorption behavior of H_2_ (or CO) under the conditions of pre-adsorbed H_2_O (or CO_2_) were systematically studied and combined with hematite reduction experiments to further advance the basic research on the reaction mechanism of hematite reduction by a H_2_-CO gas mixture, enabling the wider application of hydrogen-containing energy in the ironmaking process and promoting the development of the ironmaking industry towards a greener and more sustainable direction.

## 2. Method and Experiment

### 2.1. Computational Details

This study was based on the first-principles calculation method of the DFT and was completed by using the Cambridge serial total energy package (CASTEP) module in the Materials Studio calculation software (MS2016, BIOVIA, Vélizy-Villacoublay, France) [32]. The electron exchange and correlation energy were calculated by the Perdew–Burke–Ernzerhof (PBE) functional of the general gradient approximation (GGA) [33,34]. The Monkhorst–Pack method [35] was used to determine the value of the k-point in the Brillouin zone. The pseudopotential used in the calculation was the ultrasoft pseudopotential proposed by Vanderbilt [36]. All computations were performed in reciprocal space at 0 K, with structural optimizations implemented via the Broyden–Fletcher–Goldfarb–Shanno (BFGS) algorithm [37]. In addition, the effects of zero-point energy and dispersion correction on gas adsorption energy were neglected in the calculation process if no new chemical bonds were formed in the complex system [38,39,40,41].

The convergence test was carried out for the set of the k-point mesh density and plane-wave cutoff energy of the DFT calculations. According to the test results, the value of the k-point mesh in the bulk-phase calculation was set to 2 × 2 × 2, the value of the k-point mesh in the surface adsorption calculation was set to 2 × 2 × 1 and the plane-wave cutoff energy was set to 500 eV. In the calculation process, the convergence criterion of energy was set to 1.0 × 10^−5^ eV/atom, the convergence criterion of maximum force was fixed at 0.03 eV/Å, the convergence criterion of maximum pressure was established at 0.05 GPa, the convergence criterion of maximum displacement was defined as 0.001 Å and the value of Fermi-level smearing was applied at 0.2 eV. The adsorption energy of the given adsorption system is defined as the difference between the total energy of the adsorption system after structural relaxation and the total energy of each substance before adsorption, as shown in Equation (1), in which the larger absolute value of the adsorption energy is the more stable structure of the post-adsorption system.

(1) $$E_{\mathrm{ads}}\;=\;E_{adsorption\;system}\;-\;E_{adsorption\;substance}\;-\;E_{adsorption\;gas}$$

where $E_{\mathrm{ads}}$ represents the adsorption energy $E_{adsorption\;system}$, $E_{adsorption\;substance}$ and $E_{adsorption\;gas}$ are the total energy of the adsorption system after structural relaxation, the total energy of the adsorbed substance before adsorption and the total energy of the gas before adsorption, respectively.

**Figure 1:**
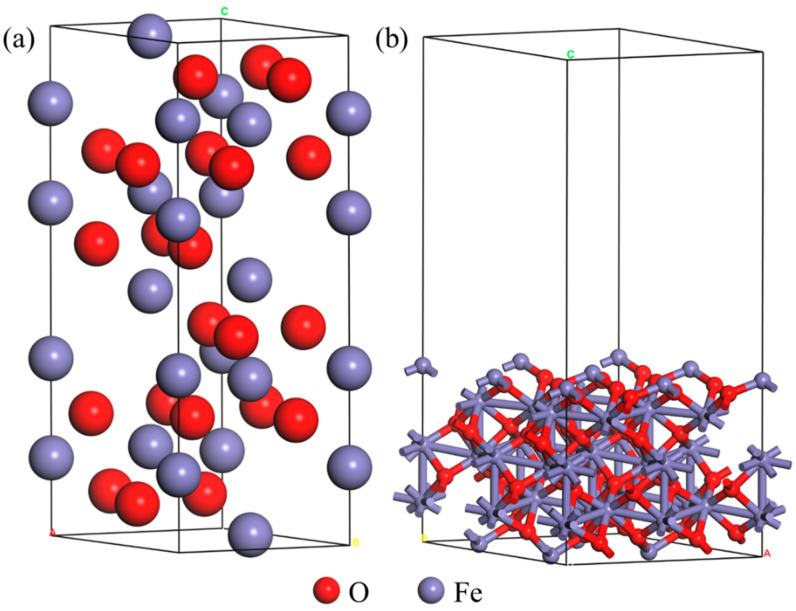
Structure diagram: (**a**) Fe_2_O_3_ crystal structure; (**b**) Fe_2_O_3_ (001) supercell model structure.

The space group of hematite (Fe_2_O_3_) is R-3C, which is a rhombohedron unit cell structure, and its bulk structure is shown in Figure 1a. According to existing relevant research [42,43,44,45], the Fe_2_O_3_ (001) surface is the low-index surface with relatively good stability and is selected as a typical research object. Therefore, in this paper, the fully relaxed Fe_2_O_3_ unit cell was cleaved to generate the initial Fe_2_O_3_ (001) surface structure for research. Subsequently, a (2 × 2 × 1) supercell model of the Fe_2_O_3_ (001) surface was constructed and a 12 Å vacuum layer was set in the *Z*-axis direction and relaxed again for the system. The results after relaxation are shown in Figure 1b.

### 2.2. Experiment Procedure

**Figure 2:**
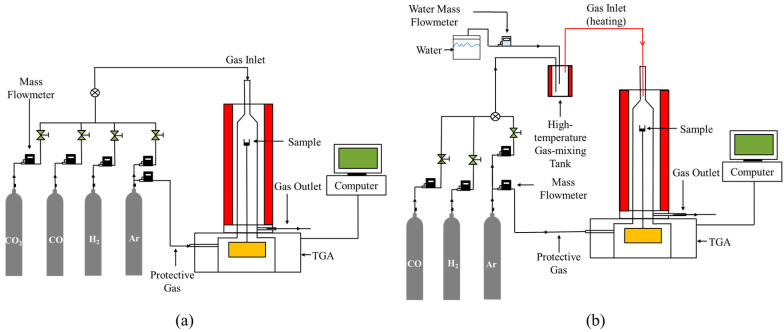
The schematic diagram of the experimental setup: (**a**) H_2_-CO-CO_2_; (**b**) H_2_-CO-H_2_O.

**Figure 3:**
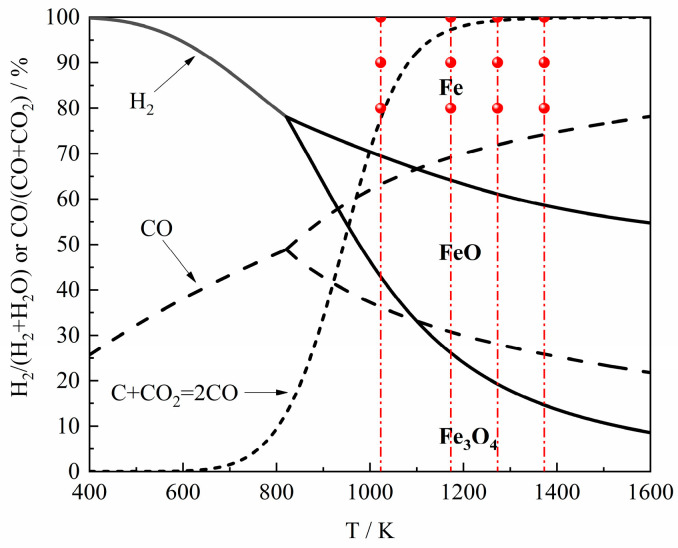
The trend of experimental conditions in this study.

Figure 2 presents the schematic diagram of the experimental setup. An amount of 100 mg of calcining reagent-grade Fe_2_O_3_ powder was loaded into an alumina crucible (inner diameter: 16 mm) and placed in a thermo-gravimetric analyzer (TGA, HCT-4, Henven, Beijing, China). The Ar (argon, PRAXAIR, Beijing, China) gas was introduced into the TGA to completely remove the air, and the temperature was increased from room temperature to the target temperature at a rate of 20 K/min under continuous Ar gas protection. The Ar gas was switched to the gas reactant to start the reduction reaction when the temperature was stable for 10 min. After the reaction was completed, the gas reactant was switched to Ar gas again, and then the sample was cooled to room temperature under the protection of Ar gas. The gases of H_2_ (99.999%), Ar (99.999%), CO (99.99%) and CO_2_ (99.99%) (PRAXAIR, Beijing, China) used in the experiment were controlled by a gas mass flowmeter (Alicat, AZ, USA, accuracy: 0.5%), and the water vapor part of the gas reactant was controlled by an appliance provided by Bronkhorst (Gelderland, The Netherland, accuracy: 0.4%). Table 1 shows the experimental conditions employed in this study, and the trend of experimental conditions is shown in Figure 3.

## 3. Results and Discussion

### 3.1. DFT Calculation

**Figure 4:**
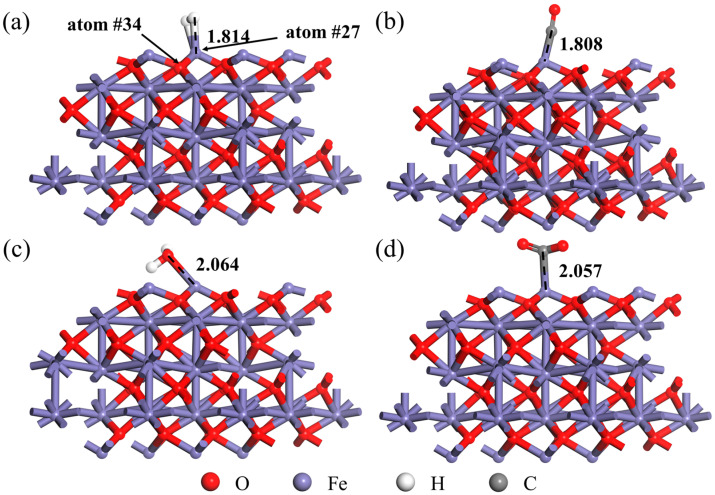
Adsorption system: (**a**) Fe_2_O_3_-H_2_; (**b**) Fe_2_O_3_-CO; (**c**) Fe_2_O_3_-H_2_O; (**d**) Fe_2_O_3_-CO_2_.

The relaxed H_2_, CO, H_2_O and CO_2_ molecules were positioned on the Fe_2_O_3_ (001) surface, respectively. The initial structure of the H_2_, CO, H_2_O and CO_2_ molecules’ adsorption on the Fe_2_O_3_ (001) surface is shown in Figure S1 (Supplementary Materials). The structure optimization was conducted by the CASTEP module of Materials Studio to obtain the stable adsorption systems, as shown in Figure 4. The situation without a stable adsorption structure is not discussed in this study. The existence of bonds between atoms in the figure does not necessarily mean that chemical bonds have been formed but rather for the convenience of displaying the distance between atoms (collectively referred to as bond length for descriptive convenience). As can be seen from Figure 4, both H atoms of the H_2_ molecule would adsorb on the Fe-top site of the Fe_2_O_3_ (001) surface (the Fe atom number in the adsorption system was 27, and the position is shown in Figure 4) where the Fe-H bond length (d_Fe-H_) is 1.814 Å. As evidenced in Figure 4b–d, it can be found that the CO, H_2_O and CO_2_ molecules all adsorbed on the Fe atom (atom #27). In the Fe_2_O_3_-CO adsorption system, the C atom in CO molecule was adsorbed to the Fe site, and the Fe-C bond length was 1.808 Å. In the Fe_2_O_3_-H_2_O adsorption system, the O atom rather than the H atom in the H_2_O molecule was adsorbed to the Fe site, and the Fe-O bond length was 2.064 Å. In the Fe_2_O_3_-CO_2_ adsorption system, the C atom in the CO_2_ molecule was adsorbed to the Fe site, and the Fe-C bond length was 2.057Å. By comparing the bond length between the atom in the adsorbed gas molecule and the adsorption site, it can be found that the distance between H_2_O and CO_2_ from the Fe_2_O_3_ (001) surface was greater than that between H_2_ and CO, but the bond length between the two adsorption atoms in different adsorption systems did not necessarily represent the stability of adsorption.

A negative value of adsorption energy for the adsorption system indicates that the energy of the adsorption system is reduced during the adsorption process. The smaller adsorption energy (the larger absolute value) indicates that the system structure is more stable after adsorption according to the second law of thermodynamics. Therefore, to obtain the stability order of each adsorption system structure in thermodynamics, the adsorption energy of each adsorption system was obtained according to Equation (1), as shown in Table 2. From Table 2, the adsorption energy of the Fe_2_O_3_-CO adsorption system was −1.317 eV, while the adsorption energy of the Fe_2_O_3_-H_2_ adsorption system was −0.013 eV, which confirmed that the Fe_2_O_3_-CO adsorption system resided in a lower energy state than the Fe_2_O_3_-H_2_ adsorption system, indicating that the Fe_2_O_3_-CO adsorption system was more stable than the Fe_2_O_3_-H_2_ adsorption system. Similarly, the adsorption energy of the Fe_2_O_3_-H_2_O adsorption system was −0.702 eV, indicating that its adsorption stability was also higher than that of the Fe_2_O_3_-H_2_ adsorption system.

In order to explore the effect of H_2_, CO, H_2_O and CO_2_ adsorption on the Fe_2_O_3_ (001) surface, the net charge and Fe-O bond of the Fe atom adsorbed by these gas molecules on the Fe_2_O_3_ (001) surface were further analyzed. The Fe atom adsorbed by the gas molecule also bonded with the three adjacent O atoms at the same time. Only one of the Fe-O bonds was selected for analysis (the O atom number in the adsorption system was 34), as the other two Fe-O bonds were consistent with the same trend. The specific parameters are shown in Table 3.

**Figure 5:**
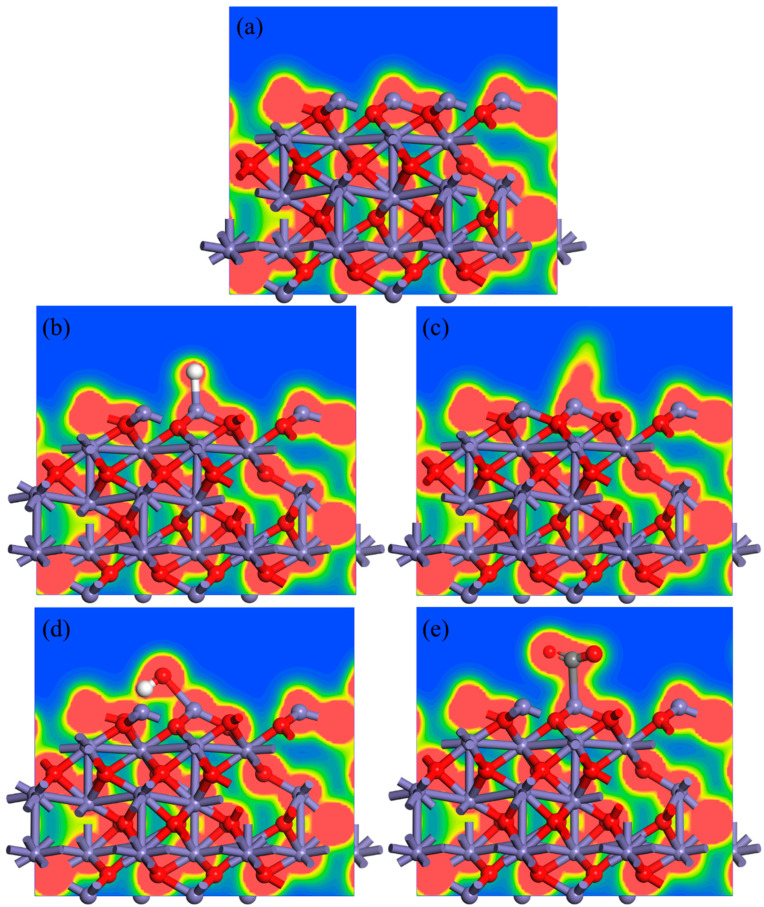
Charge density diagram of adsorption system: (**a**) Fe_2_O_3_; (**b**) Fe_2_O_3_-H_2_; (**c**) Fe_2_O_3_-CO; (**d**) Fe_2_O_3_-H_2_O; (**e**) Fe_2_O_3_-CO_2_.

It can be found from Table 3 that the bond length of the Fe-O bond was 1.716 Å when the Fe_2_O_3_ (001) surface did not adsorb the gas molecule, and once the gas molecule was adsorbed to the Fe atom site, the bond length of the Fe-O bond became longer, which means that the Fe atom moved away from the O atom after the adsorption of the gas molecule. In this study, the bond length of the Fe-O bond after the adsorption of the CO molecule was 1.772 Å, which is the longest. The bond length of the Fe-O bond was 1.758 Å when the adsorbed gas molecule was the H_2_O molecule, which was also longer than the Fe-O bond length of 1.746 Å when adsorbing the H_2_ and CO_2_ molecules. By analyzing the bond order of the Fe-O bond, it can be found that although the bond length of the Fe-O bond was the longest in the Fe_2_O_3_-CO adsorption system, the bond order of the Fe-O bond was 0.52, and the bond strength was only lower than that of the Fe-O bond without the gas molecule adsorbing. The bond order of the Fe-O bond in the Fe_2_O_3_-H_2_ adsorption system decreased to 0.49, indicating that the Fe-O bond was weaker than the Fe-O bond in the Fe_2_O_3_-CO adsorption system. The results indicate that H_2_ was stronger than CO in breaking the Fe-O bond in Fe_2_O_3_ during gas adsorption. In addition, the bond order of the Fe-O bond in the Fe_2_O_3_-H_2_O adsorption system was the smallest, which means that this was the lowest bond strength of Fe-O bond. As a result, H_2_O had the greatest influence on the Fe-O bond, while H_2_O could not continue to react with Fe_2_O_3_ due to its chemical property. It was found that when the H_2_, CO, H_2_O and CO_2_ molecules were adsorbed to the Fe atom on the Fe_2_O_3_ (001) surface, their electrons were attracted by the Fe atom and therefore increased the net charge of the Fe atom. When the adsorbed gas molecule was the H_2_ molecule, the net charge of the Fe atom increased from 0.75e to 0.95e. The CO molecule increased the net charge of the Fe atom to 0.76e, indicating that the attraction of the H_2_ molecule to electrons was greater than that of the CO molecule. The charge density diagram, as shown in Figure 5, can also indirectly confirm this result.

**Figure 6:**
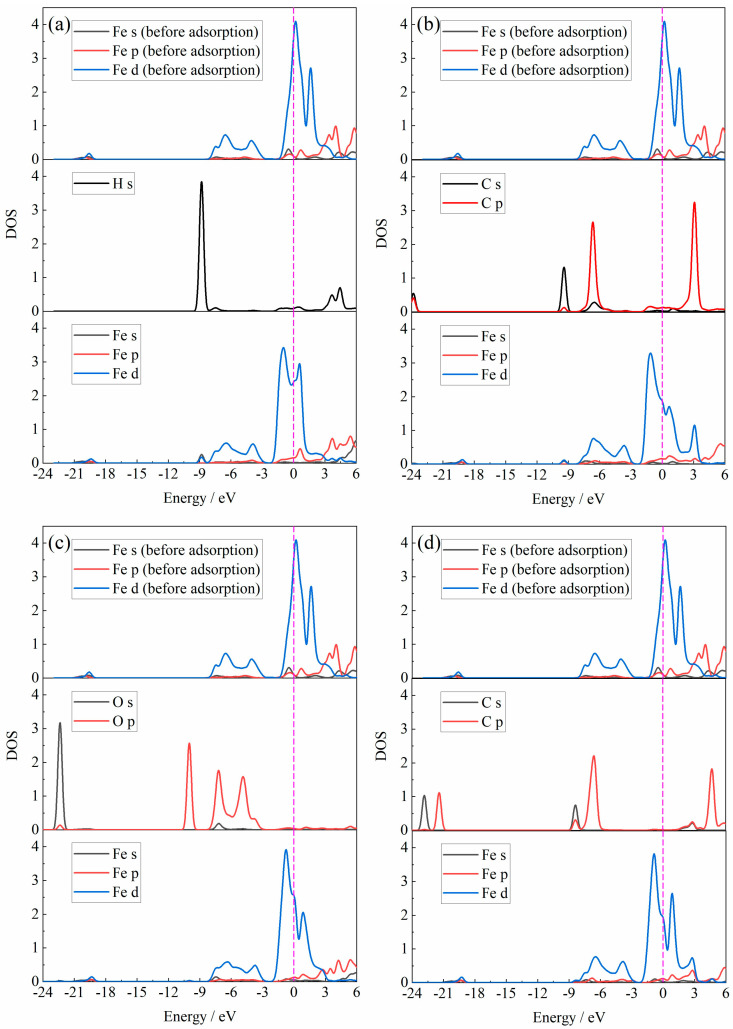
The DOS of adsorption atoms in the adsorption system: (**a**) Fe_2_O_3_-H_2_; (**b**) Fe_2_O_3_-CO; (**c**) Fe_2_O_3_-H_2_O; (**d**) Fe_2_O_3_-CO_2_.

The bonding mechanism of the adsorption of the H_2_, CO, H_2_O and CO_2_ gas molecules on the Fe_2_O_3_ (001) surface was further investigated by calculating the density of states (DOS) of the adsorbed atom in the gas molecule and the adsorbed Fe atom on the Fe_2_O_3_ (001) surface, respectively, and the results of the calculation of the DOS are shown in Figure 6.

From Figure 6, it is observed that when the H_2_ molecule was adsorbed onto the Fe_2_O_3_ (001) surface, the s-orbital of the H atom had a resonance peak with the s-orbital and d-orbital of the Fe atom at an energy of −8.8 eV, forming a covalent bond. When the CO molecule was adsorbed onto the Fe_2_O_3_ (001) surface, the C atom in the CO molecule adsorbed and bonded with the Fe atom. The s-orbital and p-orbital of the C atom formed a resonance peak with the s-orbital and d-orbital of the Fe atom at an energy of −9.3 eV. There was also an electronic interaction between the s-orbital and p-orbital of the C atom and the d-orbital of the Fe atom at an energy of −6.6 eV, the resonance of which was more obvious due to the higher DOS of the C atom and Fe atom. When the adsorption gas molecules were H_2_O and CO_2_, the DOS of the s-orbital and p-orbital of the Fe atom was relatively less obvious than that of the DOS under the adsorption conditions of the H_2_ and CO molecules. The resonance orbital of the Fe atom with the O atom and the C atom was basically the d-orbital.

**Figure 7:**
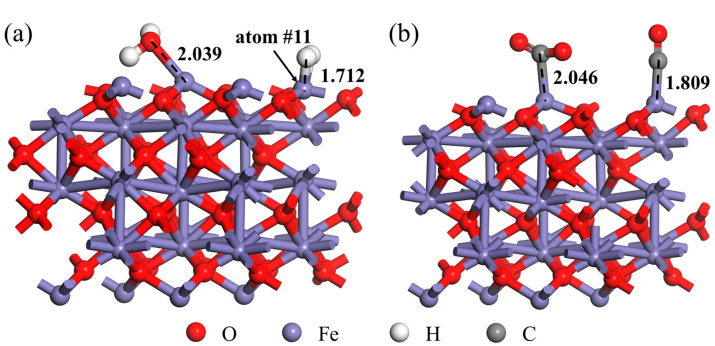
Adsorption system: (**a**) Fe_2_O_3_-H_2_O-H_2_; (**b**) Fe_2_O_3_-CO_2_-CO.

When the interfacial chemical reaction between H_2_ (or CO) and Fe_2_O_3_ occurs and generates H_2_O (or CO_2_), the adsorption of the product H_2_O (or CO_2_) on the Fe_2_O_3_ surface will affect the subsequent H_2_ (or CO) adsorption behavior. To better investigate this effect, based on the established Fe_2_O_3_-H_2_O adsorption system or Fe_2_O_3_-CO_2_ adsorption system, a H_2_ or CO molecule was further introduced into the adsorption system for co-adsorption, respectively. The initial structure of the Fe_2_O_3_-H_2_O-H_2_ and Fe_2_O_3_-CO_2_-CO system is shown in Figure S2 (Supplementary Material). The adsorption site was the equivalent Fe atom on the Fe_2_O_3_ (001) surface that was adsorbed by the H_2_ or CO described in the previous section, as shown in Figure 7.

It can be found from Figure 7 that the H_2_ molecule was stably adsorbed on the Fe_2_O_3_- H_2_O adsorption system. The distance (d_Fe-H_) between the adsorbed Fe atom (the Fe atom number in the adsorption system was 11) and the adsorbed H atom on the Fe_2_O_3_ (001) surface was 1.712 Å (as shown in Table 4), which was smaller than the d_Fe-H_ (value of 1.814 Å) when the H_2_ molecule was adsorbed on the Fe_2_O_3_ (001) surface without pre-adsorption of the H_2_O molecule. This result indicates that when the H_2_O molecule was adsorbed on the Fe_2_O_3_, the H_2_ molecule tended to be closer to the Fe_2_O_3_ surface. In addition, when the CO molecule was adsorbed on the Fe_2_O_3_-CO_2_ adsorption system, the distance between the adsorbed atoms (d_Fe-C_ value of 1.809 Å) was only slightly larger than the distance between the CO molecule directly adsorbed on the Fe_2_O_3_ (001) surface (d_Fe-C_ value of 1.808 Å), which indicates that CO_2_ had relatively little effect on the d_Fe-C_.

The adsorption energy of the H_2_ molecule adsorbed to the Fe_2_O_3_-H_2_O adsorption system was −0.132 eV, which was smaller than that of the H_2_ molecule directly adsorbed to Fe_2_O_3_ (−0.013 eV), indicating that the H_2_O molecule pre-adsorption was beneficial to the H_2_ molecule adsorption. However, on the whole, due to the presence of H_2_O molecules already absorbed on some active sites in the Fe_2_O_3_, this would affect the further reduction behavior of Fe_2_O_3_ by H_2_. The adsorption energy of the CO molecule adsorbed to the Fe_2_O_3_-CO_2_ adsorption system was −1.203 eV, which was larger than that of CO molecule directly adsorbed to Fe_2_O_3_ (−1.317 eV). Considering the adsorption energy alone, it could be found that the stability of the Fe_2_O_3_-CO adsorption system that formed when the CO molecule directly adsorbed to Fe_2_O_3_ was relatively stronger. Therefore, compared with the H_2_O molecule, CO_2_ had relatively less influence on the subsequent reduction behavior of CO with Fe_2_O_3_.

**Figure 8:**
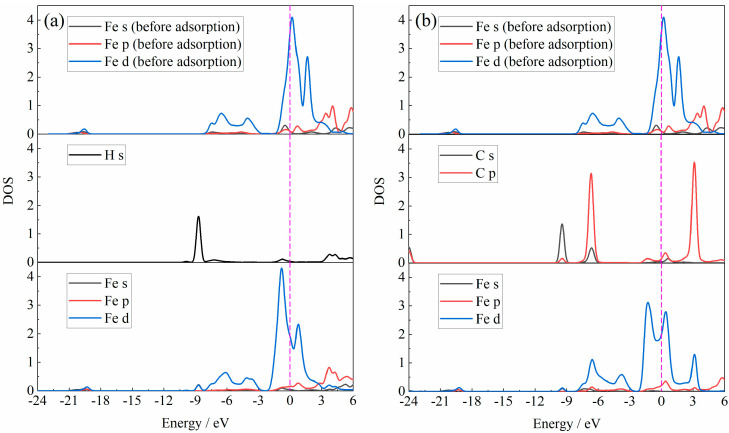
The DOS of adsorption atoms in the adsorption system: (**a**) Fe_2_O_3_-H_2_O-H_2_; (**b**) Fe_2_O_3_-CO_2_-CO.

The DOS for the adsorption atoms in the Fe_2_O_3_-H_2_O-H_2_ adsorption system and the Fe_2_O_3_-CO_2_-CO adsorption system are shown in Figure 8. Compared with Figure 6, this figured reveals that the pre-adsorption of H_2_O or CO_2_ had a minor effect on the DOS of the Fe atom at the adsorption site. The peak shape of the DOS of the Fe atom was basically similar, and the orbital resonance between the Fe and H or C atom was basically d-orbital.

### 3.2. Reduction Experiment

**Figure 9:**
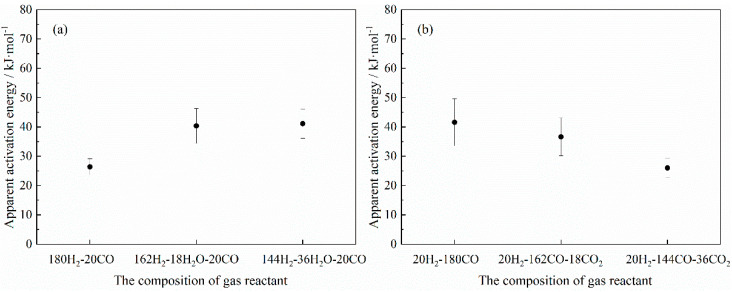
Relation between the composition of the gas reactant and the apparent activation energy: (**a**) H_2_-CO-H_2_O; (**b**) H_2_-CO-CO_2_.

The adsorption of a gas reactant on the solid reactant surface is a prerequisite for the occurrence of a gas–solid chemical reaction. The unreacted gas components in the gas mixture mainly interfere with the reaction process by affecting the diffusion or adsorption (subsequent interfacial chemical reaction) of the gas reactant. The possible rate-controlling step of the reaction (gas diffusion, interfacial chemical reaction, etc.) can be indirectly inferred from the apparent activation energy of the reaction, as shown in Table 5. In this study, the apparent activation energy for the reactions between hematite and the gas mixture was derived using the Arrhenius Equation based on previously established research and analysis methods [31,46], as shown in Figure 9.

From Figure 9a, it could be observed that the apparent activation energy of the reaction was basically around 26 kJ/mol when the H_2_-CO gas mixture was not mixed with H_2_O. Referring to the empirical relationship between the apparent activation energy and the reaction, it could be found that the possible rate-controlling step of the reaction at this time was a combined gas diffusion and interfacial chemical reaction biased towards the gas diffusion. With the addition of H_2_O in the H_2_-CO gas mixture, the apparent activation energy of the reaction increased with its content, and the possible rate-controlling step changed to a combined gas diffusion and interfacial chemical reaction biased towards the interfacial chemical reaction, which meant that the addition of H_2_O mainly influenced the occurrence of the interfacial chemical reaction. In addition, it was noteworthy that this implied that the addition of H_2_O had a relatively greater impact on the interfacial chemical reaction rather than not affecting the diffusion ability of the H_2_-CO gas mixture. However, as the CO_2_ content in the gas mixture increased, the apparent activation energy of the reaction presented a decreasing trend. When the content of CO_2_ was 20%, the apparent activation energy was decreased to 25.98 kJ/mol, and with reference to Table 5, it could be found that the possible rate-controlling step changed from a combined gas diffusion and interfacial chemical reaction biased towards the interfacial chemical reaction to a combined gas diffusion and interfacial chemical reaction biased towards gas diffusion. Therefore, comparing the effects of H_2_O and CO_2_ on the apparent activation energy of the reaction, it can be seen that the effects of H_2_O and CO_2_ on the possible rate-controlling step of the reaction were different.

According to the analysis results of the apparent activation energy of the reaction, on the whole, CO_2_ had a greater impact on the gas diffusion, while H_2_O had a greater impact on the interfacial chemical reaction (gas adsorption). Moreover, from the adsorption behavior results of the gas molecules (H_2_, CO, H_2_O and CO_2_) on the hematite surface, the adsorption energy of the Fe_2_O_3_-CO adsorption system was −1.317 eV, which was smaller than the adsorption energy of the Fe_2_O_3_-CO_2_ adsorption system (−0.076 eV), indicating that the Fe_2_O_3_-CO adsorption system was more stable than the Fe_2_O_3_-CO_2_ adsorption system. On the other hand, the adsorption energy of the Fe_2_O_3_-H_2_O adsorption system was −0.702 eV, indicating that its adsorption stability was also higher than that of the Fe_2_O_3_-H_2_ adsorption system (adsorption energy value of −0.013 eV). This suggested that in the case of competitive adsorption on the hematite surface, the H_2_O molecule would have a relatively large impact on the adsorption of the H_2_ molecule. In addition, under the conditions of pre-adsorbed H_2_O (or CO_2_), it was found that the stability of the Fe_2_O_3_-CO adsorption system formed when the CO molecule directly adsorbed to Fe_2_O_3_ was relatively stronger, and compared with the H_2_O molecule, CO_2_ had relatively less influence on the subsequent reduction behavior of CO with Fe_2_O_3_, which was consistent with the analysis results of the apparent activation energy of the reaction.

## 4. Conclusions

This study systematically investigates the adsorption behaviors of gas molecules (H_2_, CO, H_2_O and CO_2_) on hematite surface, integrated with reduction experiment results, yielding the following conclusions:

(1) The gas molecules (H_2_, CO, H_2_O and CO_2_) all adsorbed on the Fe atom of the Fe_2_O_3_ (001) surface rather than the O atom. In addition, the adsorption energy of the Fe_2_O_3_-CO adsorption system was −1.317 eV, which was smaller than the adsorption energy of the Fe_2_O_3_-H_2_ adsorption system (−0.013 eV), indicating that the Fe_2_O_3_-CO adsorption system was more stable than the Fe_2_O_3_-H_2_ adsorption system. And the adsorption energy of the Fe_2_O_3_-H_2_O adsorption system was −0.702 eV, indicating that its adsorption stability was also higher than that of the Fe_2_O_3_-H_2_ adsorption system.
(2) The H_2_O molecule pre-adsorption was beneficial to the H_2_ molecule adsorption, but the H_2_O molecule pre-adsorption affected the further reduction behavior of Fe_2_O_3_ by H_2_. However, the adsorption energy of the CO molecule adsorbed to the Fe_2_O_3_-CO_2_ adsorption system was larger than that of the CO molecule directly adsorbed to Fe_2_O_3_, indicating that the stability of the Fe_2_O_3_-CO adsorption system was relatively stronger. Considering the adsorption energy alone, compared with the H_2_O molecule, CO_2_ had relatively less influence on the subsequent reduction behavior of CO with Fe_2_O_3_.
(3) With the addition of H_2_O in the H_2_-CO gas mixture, the apparent activation energy of the reduction reaction increased with its content, which meant that the addition of H_2_O mainly influenced the occurrence of the interfacial chemical reaction. However, the apparent activation energy presented a decreasing trend as the CO_2_ content in the H_2_-CO gas mixture increased, suggesting that the possible rate-controlling step changed to a combined gas diffusion and interfacial chemical reaction biased towards gas diffusion. Therefore, on the whole, CO_2_ had a greater impact on the gas diffusion, while H_2_O had a greater impact on the interfacial chemical reaction (gas adsorption), which was consistent with the DFT calculation results.

## Figures and Tables

**Table 1:** Experimental conditions.

**H_2_:(CO + CO_2_)**	**CO:CO_2_**	**No.**	**Temperature/K**	**Flow Rate/(mL/min)**		
				**H_2_**	**CO**	**CO_2_**
1:9	10:0	1	1023	20	180	0
		2	1173	20	180	0
		3	1273	20	180	0
		4	1373	20	180	0
	9:1	5	1023	20	162	18
		6	1173	20	162	18
		7	1273	20	162	18
		8	1373	20	162	18
	8:2	9	1023	20	144	36
		10	1173	20	144	36
		11	1273	20	144	36
		12	1373	20	144	36
**(H_2_ + H_2_O):CO**	**H_2_:H_2_O**	**No.**	**Temperature/K**	**Flow Rate/(mL/min)**		
				**H_2_**	**CO**	**H_2_O**
9:1	10:0	13	1023	180	20	0
		14	1173	180	20	0
		15	1273	180	20	0
		16	1373	180	20	0
	9:1	17	1023	162	20	18
		18	1173	162	20	18
		19	1273	162	20	18
		20	1373	162	20	18
	8:2	21	1023	144	20	36
		22	1173	144	20	36
		23	1273	144	20	36
		24	1373	144	20	36

**Table 2:** Adsorption energy of adsorption system.

Adsorption System	Fe_2_O_3_-H_2_	Fe_2_O_3_-CO	Fe_2_O_3_-H_2_O	Fe_2_O_3_-CO_2_
adsorption energy/eV	−0.013	−1.317	−0.702	−0.076

**Table 3:** Parameters of Fe_2_O_3_ after adsorption of gas molecule.

Adsorption System	Fe_2_O_3_ (Initial)	Fe_2_O_3_-H_2_	Fe_2_O_3_-CO	Fe_2_O_3_-H_2_O	Fe_2_O_3_-CO_2_
d_Fe-adsorption atom_/Å	-	1.814	1.808	2.064	2.057
d_Fe-O_/Å	1.716	1.746	1.772	1.758	1.746
net charge of Fe/e	0.75	0.95	0.76	0.9	0.96
bond order of Fe-O	0.54	0.49	0.52	0.47	0.5

**Table 4:** Parameters of adsorption system.

Adsorption System	Fe_2_O_3_-H_2_	Fe_2_O_3_-H_2_O-H_2_	Fe_2_O_3_-CO	Fe_2_O_3_-CO_2_-CO
d_Fe-adsorption atom_/Å	1.814	1.712	1.808	1.809
d_Fe-O_/Å	1.746	1.763	1.772	1.772
net charge of Fe/e	0.95	0.93	0.76	0.82
bond order of Fe-O	0.49	0.48	0.52	0.49
adsorption energy/eV	−0.013	−0.132	−1.317	−1.203

**Table 5:** Relationship between apparent activation energy and possible rate-controlling step and data from Reference [47].

NO.	Apparent Activation Energy (kJ/mol)	Possible Rate-Controlling Step
1	8~16	Gas diffusion
2	29~42	Combined gas diffusion and interfacial chemical reaction
3	60~67	Interfacial chemical reaction
4	>90	Solid-state diffusion

## Data Availability

The original contributions presented in this study are included in the article/Supplementary Material. Further inquiries can be directed to the corresponding authors.

## Supplementary Materials

The following supporting information can be downloaded at https://www.mdpi.com/article/10.3390/ma18174175/s1, Figure S1: The initial structure of the (a) Fe_2_O_3_-H_2_, (b) Fe_2_O_3_-CO, (c) Fe_2_O_3_-H_2_O and (d) Fe_2_O_3_-CO_2_ systems. Figure S2: The initial structure of (a) Fe_2_O_3_-H_2_O-H_2_ and (b) Fe_2_O_3_-CO_2_-CO.
