# Supplementary material for: The Effect of H2O and CO2 on the Adsorption Behavior of H2 and CO on Hematite
Source: Materials (Basel). 2025 Sep 5;18(17):4175. doi: 10.3390/ma18174175 (PMC12430173; doi:10.3390/ma18174175)
Supplement: Supplementary file 1 [file materials-18-04175-s001.zip › materials-3820906-supplementary.pdf]

Supporting Information

The Effect of H_2O and CO_2 on the Adsorption Behavior of H_2 and CO on Hematite

Xudong Mao [1, 2, 3, #], Baoqing Zhou [1, 2, #], Hui Deng [1], Qiong Zeng [1], Jingbo Li [1], Jie Chen [1, 2, *], Yiyu Xiao [3, *] and Kuochih Chou [3]

1) Jiangxi General Institute of Testing and Certification, Nanchang, 330052, China.
2) School of Resources & Environment, Nanchang University, Nanchang, 330031, China.
3) State Key Laboratory of Advanced Metallurgy, University of Science and Technology Beijing, Beijing, 100083, China.
*Correspondence: author: *Jie Chen*, *Yiyu Xiao*
#Co-first author: Xudong Mao, Baoqing Zhou

Compared with the figures after adsorption in the paper, there are only minor differences in the apparent structure such as bond length.

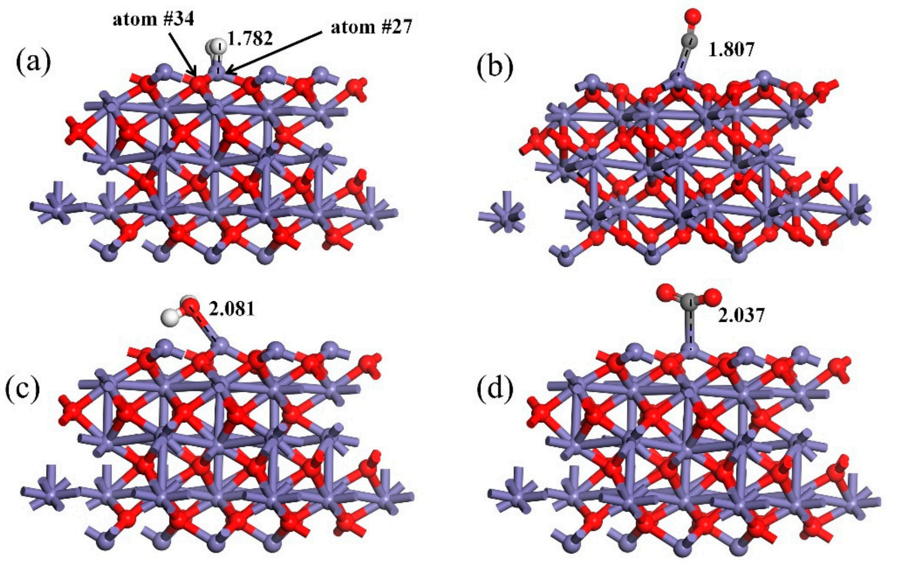

Figure S1 The initial structure of (a)Fe_2O_3-H_2, (b) Fe_2O_3-CO, (c) Fe_2O_3-H_2O, (d) Fe_2O_3-CO_2 systems.

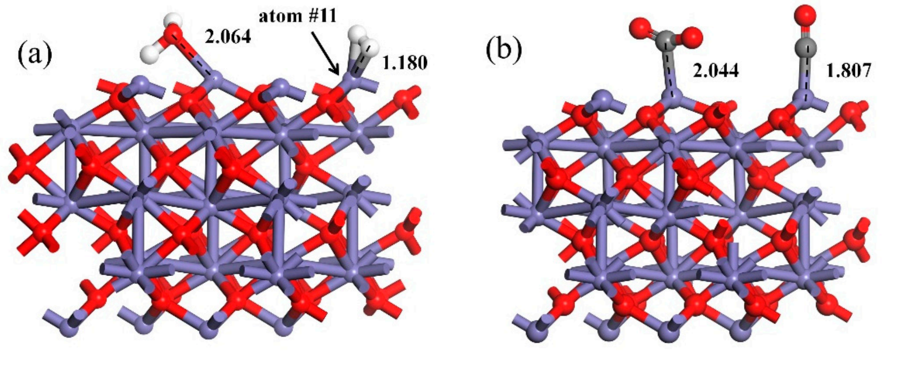

Figure S2 The initial structure of (a)Fe_2O_3-H_2O-H_2, (b) Fe_2O_3-CO_2-CO.
